# Incidence and management of Osgood–Schlatter disease in general practice: retrospective cohort study

**DOI:** 10.3399/BJGP.2021.0386

**Published:** 2022-02-22

**Authors:** Guido J van Leeuwen, Evelien IT de Schepper, Michael S Rathleff, Patrick JE Bindels, Sita MA Bierma-Zeinstra, Marienke van Middelkoop

**Affiliations:** Department of General Practice, Erasmus University Medical Center, Rotterdam, the Netherlands.; Department of General Practice, Erasmus University Medical Center, Rotterdam, the Netherlands.; Center for General Practice, Aalborg University; Department of Health Science and Technology, Aalborg University, Aalborg, Denmark.; Department of General Practice, Erasmus University Medical Center, Rotterdam, the Netherlands.; Department of General Practice, Erasmus University Medical Center, Rotterdam, the Netherlands.; Department of General Practice, Erasmus University Medical Center, Rotterdam, the Netherlands.

**Keywords:** general practice, incidence, management, Osgood-Schlatter disease, primary health care

## Abstract

**Background:**

Osgood–Schlatter disease (OSD) is a non-traumatic knee problem that is primarily observed in sports-active children and adolescents aged 8–15 years.

**Aim:**

To determine the incidence of OSD and to gain an insight into the management of children and adolescents with OSD in general practice.

**Design and setting:**

A retrospective cohort study was conducted using a healthcare database containing full electronic health records of over 200 000 patients in general practice in and around the Dutch city of Rotterdam.

**Method:**

Patients with a new diagnosis of OSD from 1 January 2012 to 31 December 2017 were extracted using a search algorithm based on International Classification of Primary Health Care coding and search terms in free text. Data on the management of OSD were manually interpreted.

**Results:**

The mean incidence over the study period was 3.8 (95% confidence interval [CI] = 3.5 to 4.2) per 1000 person–years in those aged 8–18 years. Boys had a higher incidence rate of 4.9 (95% CI = 4.3 to 5.5) compared with girls (2.7, 95% CI = 2.3 to 3.2). Peak incidence was at 12 years of age for boys and 11 years for girls. Advice was the most commonly applied strategy (55.1%), followed by rest (21.0%), referral for imaging (19.5%), and physiotherapy (13.4%).

**Conclusion:**

To the authors’ knowledge, for the first time the incidence of OSD has been calculated using GP electronic medical files. There is a discrepancy, especially for imaging and referral to a medical specialist, between the current Dutch general practice guidelines and how GPs actually manage the condition in clinical practice.

## INTRODUCTION

Knee pain (32%) is the most frequently reported musculoskeletal pain complaint during adolescence.^1^ Knee pain accounts for the second highest number of general practice consultations for musculoskeletal problems in children and adolescents aged 3–19 years.^2^ Peak prevalence for general practice consultation because of knee pain occurs in those aged 12–17 years for children and adolescents in the mentioned age group.^2^ In Dutch general practice the incidence of consultations as a result of knee pain range from 10.6 to 17.4 per 1000 patients aged 4–24 years. In this age group, prevalence ranges from 12.4 to 22.5 per 1000 patients.^3^ Up to 25% of the children and adolescents with musculoskeletal problems will experience chronic or recurrent pain, making initial management important.^4^^–^^6^

One of the most common non-traumatic knee problems in children and adolescents is Osgood–Schlatter disease (OSD).^3^^,^^7^ According to a retrospective questionnaire distributed in an outpatient sports clinic, OSD affects 12% of the boys and girls aged 9–15 years, with an even higher prevalence in sports-active adolescents (21%).^8^ OSD is widely accepted as a traction apophysitis of the tibial tuberosity caused by repetitive strain on the patellar tendon at the point of insertion.^9^^–^^15^ OSD is often characterised by knee pain and tenderness at the tibial tuberosity, swelling, thickening of the patellar tendon, and enlargement of the tibial tuberosity during physical examination.^16^^–^^18^ The pain typically occurs during activities that load the knee extensors and subsides when they stop the activity.^16^^,^^19^^–^^21^ OSD is generally considered a self-limiting disease that is expected to cease with skeletal maturity and rarely lasts >18 months before resolving completely.^22^ However, more recent research has indicated that OSD may become chronic.^5^^,^^6^^,^^23^^–^^26^

Currently, there is a glaring absence in knowledge about OSD in general practice. Until now, most studies have focused on musculoskeletal or knee problems in general, are open population surveys, and are often not in the age range where OSD is mostly observed. Despite the common occurrence of OSD in children and adolescents, the actual consultation rate in general practice is still unclear and, to the authors’ knowledge, the initial management has never been described.^27^ The objective of this study was to estimate the age- and sex-specific incidence of OSD in Dutch general practice and to gain insight into the different types of management strategies used by GPs.

## METHOD

### Design and setting

A retrospective cohort study was conducted using the Rijnmond Primary Care Database (RPCD). The database contains anonymous longitudinal data on demographics, symptoms and diagnosis, correspondence to and from secondary care, and drug prescriptions for over 200 000 participants in the greater area of the city of Rotterdam.^28^ The RPCD is a regional derivative of the nationwide Integrated Primary Care Information database.^29^^,^^30^ In the Netherlands, registration with a GP is obligated for citizens and if medical care is required the GP is contacted first, including for referrals to secondary care.

**Table 1:** How this fits in

Osgood–Schlatter disease (OSD) is a non-traumatic knee problem that mainly presents in children and adolescents during puberty and has a significant negative effect on quality of life, physical activity, and social interactions. This study, to the authors’ knowledge, is the first retrospective cohort study that provides an insight into the incidence of OSD in general practice as well as the management strategies used by GPs. The study found a discrepancy between the Dutch guideline for GPs and the described management. There was greater use of imaging and referral to a medical specialist compared with guideline recommendations. Better understanding and management of OSD by GPs could decrease the number of consultations and thus the overutilisation of imaging and referral to a medical specialist.

### Study cohort

The study population consisted of children and adolescents aged 8–18 years with a new episode of OSD between 1 January 2007 and 31 December 2017. The diagnosis was considered new if the patient had not been diagnosed with OSD in the preceding 18 months before initial diagnosis after their start date in the database. Patients could be included in the study population more than once, if there was >18 months between the initial diagnosis and subsequent consultations for OSD. Diagnoses of OSD were identified using International Classification for Primary Care (ICPC) coding and with supporting keywords in the free text.^31^

Patients were considered an ‘OSD case’ if they received the ICPC code L94 (Osgood–Schlatter/other osteochondropathy), L94.02 (Osgood–Schlatter Disease), or if they received the ICPC code L15 (Knee symptoms/complaint) in combination with the words ‘Osgood–Schlatter’, ‘Osgood’, or ‘Schlatter’ in the free text of the consultation with the GP. The final algorithm excluded hits that were combined with terms of negation (ex., not, or no) and hits where the GP combined the L94 and L94.02 ICPC codes with a specific diagnosis other than OSD, for example, ‘patellofemoral pain’ or ‘Sever’s disease’. Individuals with cases were considered to be a true OSD case if the GP defined the consultation as OSD or considered it as one of several possible diagnoses (that is, in the differential diagnosis). In this study, cases where the OSD diagnosis did not match these definitions were excluded from further analyses. Unclear cases were inspected by a senior researcher (the second author), and final decisions and whether or not to include the case as OSD were based on consensus.

### Data extraction

The full medical files were examined from the consultation date of the initial diagnosis until the end date of the medical file in the database. For each patient, information on date of birth and sex were extracted. Furthermore, the interventions applied by the GP, the referrals, and the number of consultations with the GP for one episode of complaints related to OSD were extracted from the full medical record by one of the authors (the first author).

The management strategies that took place at every consultation were registered: advice, wait and see, rest, medication, or cooling therapy; and referral comprising imaging (X-ray, ultrasonography, or magnetic resonance imaging), physiotherapist, orthopaedic surgeon, sports medicine, and other (para)medical specialists (that is, podiatry or therapeutic movement therapy). Even though advice, wait and see, and rest often are interchangeable and have the same intention, differentiation was made based on the use of specific words in the text. Multiple management strategies could be administered per consultation.

For each patient the date of the first consultation and each subsequent consultation were extracted as well as the total number of consultations. Telephone consultations were counted as consultations, provided that the GP registered a contact with the patient and discussed the diagnosis or management of OSD.

### Statistics

The incidence rate of OSD per age group from 8–18 years was determined by dividing the number of OSD cases by the total number of person–years of follow-up in all patients aged 8–18 years included in the RPCD and expressed per 1000 person–years. The 95% confidence intervals (CIs) were calculated using Poisson distribution. The incidence rate was calculated over the period from 1 January 2012 to 31 December 2017, as there was a low number of participating GP practices in the database before 2012 and numbers are therefore more robust by using data from 2012 onwards. Descriptive statistics were used to describe the management strategies applied and number of consultations for patients with OSD in the entire study period.

All analyses were performed using IBM SPSS Statistics (version 25), R Studio was used for Poisson distribution.

## RESULTS

### General characteristics

**Figure 1.**
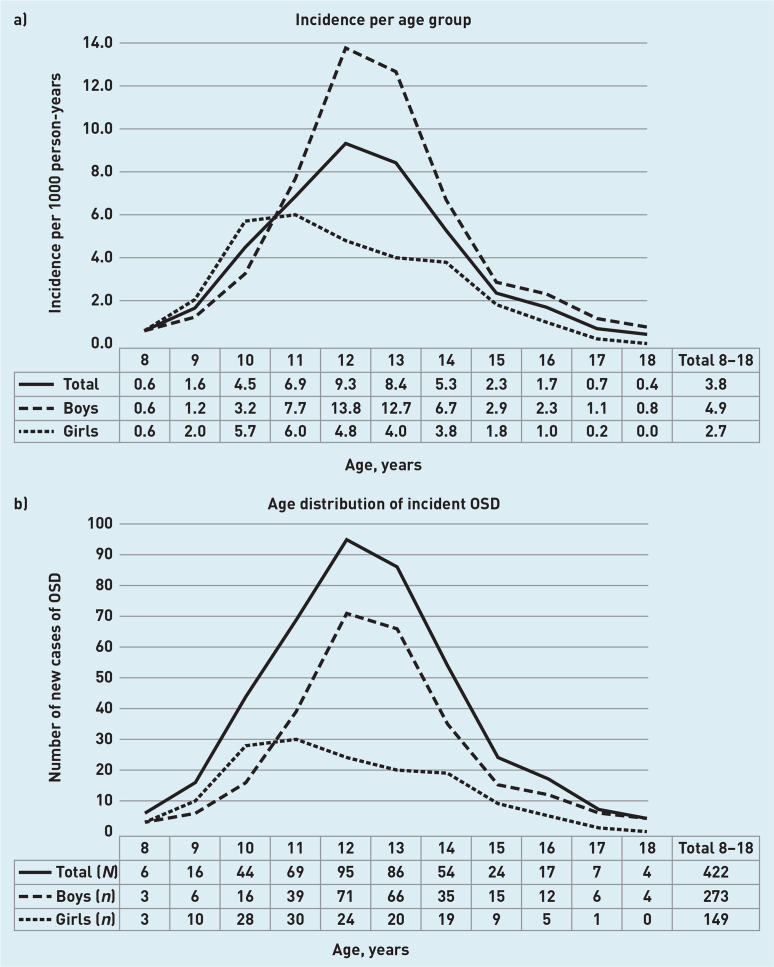
*Incidence and age distribution of Osgood–Schlatter disease (OSD) during the years 2012–2017.* *a) The incidence of OSD per age group. b) Age distribution in a cohort of 422 patients with incident OSD.*

The search strategy in the database delivered 716 OSD cases, containing 556 patients aged 8–18 years at the time of diagnosis. After manual inspection, 489 true patients with OSD remained, including 515 new OSD episodes. The overall incidence in this population was 3.8 (95% CI = 3.5 to 4.2) per 1000 person–years. A total of 64.7% of patients with OSD were boys, with an incidence of 4.9 (95% CI = 4.3 to 5.5) per 1000 person–years compared with 2.7 (95% CI = 2.3 to 3.2) in girls (Figure 1).

Within the period of 2012 to 2017, the yearly incidence was stable with overlapping 95% CIs. The median age of the incident OSD population was 12 years (P25–75 11–13 years), the median age for boys was 12 years (P_25–75_ 11–14 years), and girls 11 years (P_25–75_ 10–13 years). Peak incidence was 13.8 (95% CI = 10.8 to 17.4) per 1000 person–years at age 12 for boys and 6.0 (95% CI = 4.1 to 8.6) at age 11 for girls (Figure 1).

### Consultations and management strategies

The 515 incident cases resulted in a total of 724 consultations (range 1 to 4). In 69.3% of incident cases (*n* = 357), the OSD episode was limited to one consultation.

Details about the provided management strategies during the consultations are shown in Supplementary Table S1. Over all consultations, including follow-up consultations, advice was the most commonly applied strategy (55.1%, *n* = 399/724), followed by rest (21.0%, *n* = 152/724), and referral for imaging (19.5%, *n* = 141/724) and physiotherapy (13.4%, *n* = 97/724).

During the first consultation of all episodes, advice was the most frequently applied management strategy by the GP (61.0%, *n* = 314/515), followed by rest (23.5%, *n* = 121/515), and imaging (17.5%, *n* = 90/515). In 10.9% (*n* = 56/515) the GP applied a wait-and-see policy. Analgesics (either paracetamol or non-steroidal inflammatory drugs) were prescribed in 6.8% (*n* = 35/515) and cooling therapy in 3.5% (*n* = 18/515) of the first consultations. Referral was most often to a physiotherapist (11.5%, *n* = 59/515) followed by the orthopaedic surgeon (3.3%, *n* = 17/515), sports physician (1.0%, *n* = 5/515), and other (para)medical specialists in 1.6% (*n* = 8/515) of first consultations.

### Follow-up consultations

In total, 158 (30.7%) incident cases had multiple consultations (range 2 to 4) for OSD with their GP within 18 months of the initial diagnosis: 59.5% were boys with a median age of 12 (P25–75 12–13 years). The median time between the first and second consultation in these patients was 53 days (P25–75 13–165 days).

Supplementary Table S1 shows that advice is the most often applied management strategy until the fourth consultation when referral to the orthopaedic surgeon was most often applied. In general, the rate of referral for imaging or to a medical specialist rose with each following consultation. For example, almost a third of patients (27.7%) at second consultation were referred for imaging. Furthermore, the rate of referral to the orthopaedic surgeon increased with each following consultation, that is, a rate of 3.3% at first and 44.4% at fourth consultation.

**Figure 2.**
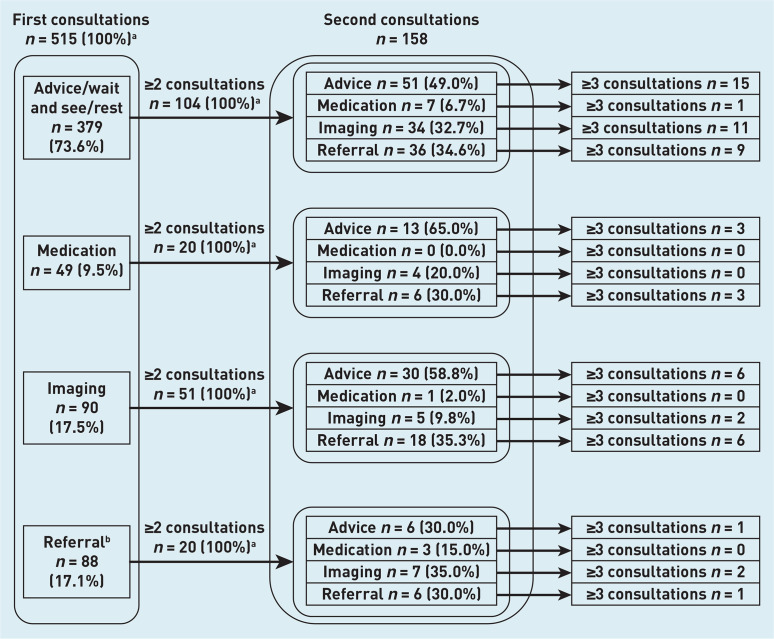
*Overview of patients and types of management depending on type of management at first consultation.* *^a^****Patients could receive >1 intervention per consultation, therefore the total of patients in these cells does not add up to 100%.*** *^b^****Referral to the physiotherapist, orthopaedic surgeon, sports physician, or other (para) medical specialists.***

Figure 2 provides an overview on the flow of patients after their first consultation. Patients that received medication or were referred for imaging had a higher percentage of second consultations, respectively 40.8% (*n* = 20/49) and 56.7% (*n* = 51/90), compared with advice and referral (27.4% [*n* = 104/379] and 22.7% [*n* = 20/88]). Irrespective of the applied management at first consultation, the amount of advice given at second consultation remained relatively stable whereas the use of imaging and rate of referral rose.

## DISCUSSION

### Summary

To the authors’ knowledge, this study is the first retrospective cohort study that provides insight into the incidence of OSD in general practice as well as the different management strategies applied in this population based on longitudinal population data. An incidence of OSD of 3.8 per 1000 person–years in those aged 8–18 years was found, with a higher incidence for boys than girls. This incidence indicates that an average GP practice (255 patients aged 8–18 years total) in the Netherlands sees about one new patient with OSD per year.

In the population studied, the peak incidence was 1 year earlier in girls compared with boys. Most patients (69.3%) only visited the GP once. Most patients were managed with advice and rest at first consultation. Nevertheless, in 17.5% of the cases the GP referred the patient for imaging at first consultation despite the advice of the current Dutch general practice (NHG) guideline.^32^ Only 11.5% were referred to a physiotherapist.

### Strengths and limitations

To the authors’ knowledge, this is the first study to examine the incidence of OSD based on medical files for a large group of patients in general practice. It gives a representative overview on the incidence and management in general practices.

GP medical records are not primarily intended for data collection and have certain limitations, for example, possible selection bias as a result of the diagnostic accuracy of the GP and dependence on reporting in the medical file by the GP.^33^ Moreover, technically speaking the incidence may be considered as the incidence of recorded consultations, as this study was dependent on medical files. To limit the possible underestimation of the overall incidence because of limited medical notes or non-uniform ICPC coding, multiple ICPC codes and free-text terms to identify patients with OSD were used.

In this study a maximum duration of complaint cut-off point of 18 months was used.^22^ In the analyses this may have led to a duplicate count of one OSD incident into two separate incidents. This could, for example, lead to a patient being referred to the orthopaedic surgeon at first consultation whereas this actually was the third consultation within a larger timeframe. The authors believe this had limited impact on the overall conclusions as this situation could only have occurred in a maximum of 26 patients. The decision to maintain the cut-off point of 18 months came from the most common belief that OSD, aside from rare instances, is not a complaint that spans multiple years.^22^

Lastly, 123 (23.9%) incident cases did not have at least 18 months of follow-up time. However, this is highly unlikely to have a relevant impact on the results as most patients (91.8%) had a maximum of two consultations with a median of 53 days between first and second consultation.

### Comparison with existing literature

An incidence of 4.9 and 2.7 for boys and girls per 1000 person–years, respectively, was found. Currently, to the authors’ knowledge, no other incidence data for OSD are available in the literature. Similar to the findings in the current study, a previous study also showed a higher frequency of OSD in boys (15.2%) compared with girls (10.0%) in children aged 9–15 years.^8^ In the population in the current study, the difference in peak incidence between girls and boys was only 1 year, at 11 and 12 years of age, respectively. This is in concordance with previous literature where the occurrence of OSD was higher in boys compared with girls, but complaints presented at an earlier age in girls. This is possibly because of the earlier onset of puberty and skeletal maturation in girls.^8^^,^^16^^,^^34^

According to current research and the NHG guideline, OSD is a clinical diagnosis based on anamnesis and physical examination, and the management of OSD is conservative, guided by the severity of the symptoms.^19^^,^^22^^,^^32^^,^^35^^,^^36^ A multinational survey showed that healthcare professionals favour education and exercise therapy in the management of OSD.^27^ In concordance with previous literature and the NHG guideline^32^ most patients in this study received advice (55.1%) followed by rest (21.0%). However, most adolescents with OSD are often highly physically active, thus resting and therefore recovering may be difficult for patients.^8^^,^^37^

Recently, Rathleff *et al* investigated the effectiveness of an intervention consisting of education on activity modification and knee-strengthening exercises in 51 adolescents with OSD.^38^ They showed that patella tendon loading and pain management, hip and knee strengthening, and jumping exercises resulted in a self-reported improvement of 80% and 90% after 12 to 52 weeks, respectively. In the current study, patients were referred to a physiotherapist in only 13.4% of all consultations, although the actual content of the management is unknown. Despite limited evidence from the literature, personalised physical activity programmes may offer a beneficial alternative management approach to improve clinical outcomes.

The NHG guideline^32^ explicitly states that there is no added benefit whatsoever of imaging (X-ray or magnetic resonance imaging) and referral to an orthopaedic surgeon.^39^ Remarkably, overall, patients were referred for imaging or to an orthopaedic surgeon in almost 20% and 8% of all consultations, respectively. This discrepancy could be because of a lack of knowledge regarding OSD and the management of OSD by the GP, in addition to an attempt at gaining insight into the severity of the complaint or to appease the patient. Lastly, it may be a sign of the growing overutilisation of diagnostic imaging.^40^ The rate of referral to an orthopaedic surgeon seems to correlate with the number of consultations, as the percentage increased with each following consultation, that is, a rate of 3.3% at the first consultation and 44.4% at the fourth consultation. This is likely explained by the severity of the complaint; patients with more severe OSD will probably visit the GP more often for the complaint. Furthermore, the ineffectiveness of previous advice or management can also contribute to the GP referring to the orthopaedic surgeon to get an expert opinion.

### Implications for research and practice

OSD is a common and impactful^37^ complaint in children and adolescents with an incidence of 3.8 per 1000 person–years. Peak incidence for boys and girls was at age 12 and 11 years, respectively. There is a discrepancy between the current NHG guideline and how GPs manage the condition in clinical practice. As OSD mostly occurs in sports-active adolescents,^8^^,^^37^ future research into personalised physical activity programmes may offer opportunities to make progress in effectively treating OSD in general practice. Better understanding and management of OSD, specifically for persistent cases, could decrease the number of consultations and thus the overutilisation of imaging and referral to a medical specialist.
